# High performance surface-modified TiO_2_/silicone nanocomposite

**DOI:** 10.1038/s41598-017-05166-7

**Published:** 2017-07-20

**Authors:** Pei Huang, Han-Qiao Shi, Hong-Mei Xiao, Yuan-Qing Li, Ning Hu, Shao-Yun Fu

**Affiliations:** 1 College of Aerospace Engineering, Chongqing University, Chongqing, 400044 China; 2 Aerospace Research Institute of Materials & Processing Technology, Beijing, 100076 China; 3 Technical Institute of Physics and Chemistry, Chinese Academy of Sciences, Beijing, 100190 China

## Abstract

The mismatch of refractive index (RI) between light emitting diode (LED) chips and packaging resins severely lowers the lighting emitting efficacy of LED. The RI can be enhanced by the introduction of high RI nanoparticles but meanwhile it is a great challenge to maintain the high transparency for resins due to the agglomeration of nanoparticles. In this work, a facile strategy is proposed to fabricate silicone nanocomposites with a high transparency (>88%, less than 2% decrease relative to pure silicone resin), largely enhanced RI (an increase from 1.42 to 1.60) and improved thermal stability (73 °C increase in weight loss of 50%). Specifically, the ultra-fine monodispersed TiO_2_/silicone composites are prepared by direct solvent mixing of 1 wt% surface modified TiO_2_ nanoparticles (S-TiO_2_) into the silicone resin, in which S-TiO_2_ are prepared by direct introduction of titanate coupling agent in the process of TiO_2_ growth to induce the formation of protective layer on the surfaces of TiO_2_ nanoparticles. This methodology demonstrated is simple, cost-effective and versatile for the massive fabrication of highly transparent LED packaging materials with greatly enhanced refractive index and meanwhile enhanced thermal stability.

## Introduction

Light emitting diodes (LEDs) have drawn considerable interests in the last several decades due to their high lighting efficiency and lighting wavelength tunability^1–4^. In a typical LED device, lighting chip and packaging material are the main components which determine the light emitting efficacy. Concerning the long-term service of LEDs, transmittance, refractive index (RI) and thermal stability are the key criteria required for packaging materials. Silicone resin is endorsed as the first choice over other types of materials (such as epoxy resin, polymethyl methacrylate, etc.) due to its high thermal stability and facile fabrication process^5–7^. However, the RI mismatch between the lighting chip and silicone resin leads to severe light reflection at the interface of LED chip and silicone resin, and thus greatly lowers the light emitting efficacy^8^. Great efforts have been made to increase the RI of silicone resin by introduction of functionalized atoms and side chains, or bottom-up design of polymer structure^9–14^. Currently, commercial silicone resins with relatively high RI are available^15^, whereas their very high prices and complicated processing procedures hinder their wide applications as LED packaging materials.

According to the Maxwell–Garnett effective medium theory^16^, the incorporation of inorganic filler with high RI can increase the RI of the polymer matrix^8^. TiO_2_ with a high RI (n = 2.45 and 2.7 for anatase and rutile phase, respectively) and a very low absorption coefficient in the visible range is highly attractive as a filler for the fabrication of high RI packaging materials^17, 18^. However, the poor dispersion of nanoparticles in the polymer matrix is a great challenge due to their high surface energy. Although various methods have been developed to improve the dispersion of TiO_2_ nanoparticles in silicone resin, likewise, surface modification of TiO_2_ nanoparticle in the process of sol-gel^19–21^, modification of silicone resin and etc.^22, 23^. However, these approaches usually involve toxic agents and complicated processing procedures of synthesis, purification, mixing of TiO_2_ nanoparticles^24^, and so on. Therefore, a simple yet environmental-friendly approach is strongly required to fabricate highly transparent silicone nanocomposites with high RI and high thermal stability.

Recently, a facile approach to prepare size-controllable ZnO nanoparticle has been reported by the introduction of coupling agent into precursor^24, 25^. In this work, a similar strategy has been applied to synthesize surface modified ultra-fine TiO_2_ (S-TiO_2_) nanoparticles through the introduction of titanate coupling agent in the process of TiO_2_ nanoparticle growth. The protective layer formed on the surfaces of TiO_2_ nanoparticles can effectively prevent the growth of TiO_2_ nanoparticles and significantly improve the compatibility between TiO_2_ nanoparticles and the silicone resin. In principle, the introduction of inorganic TiO_2_ can enhance the thermal stability of polymers. Thus, a highly transparent TiO_2_/silicone nanocomposite with a high RI and high thermal stability can be obtained by directly mixing the ultra-fine S-TiO_2_ nanoparticles with the silicone resin. To evaluate their potential as LED packaging material, the transmittance, RI and thermal stability of the as-prepared silicone nanocomposite are examined. As a control, several types of TiO_2_ nanoparticles without the introduction of coupling agent and the corresponding composites are prepared and compared with the surface modified S-TiO_2_ case.

## Results and Discussions

### Synthesis of S-TiO_2_

**Figure 1:**
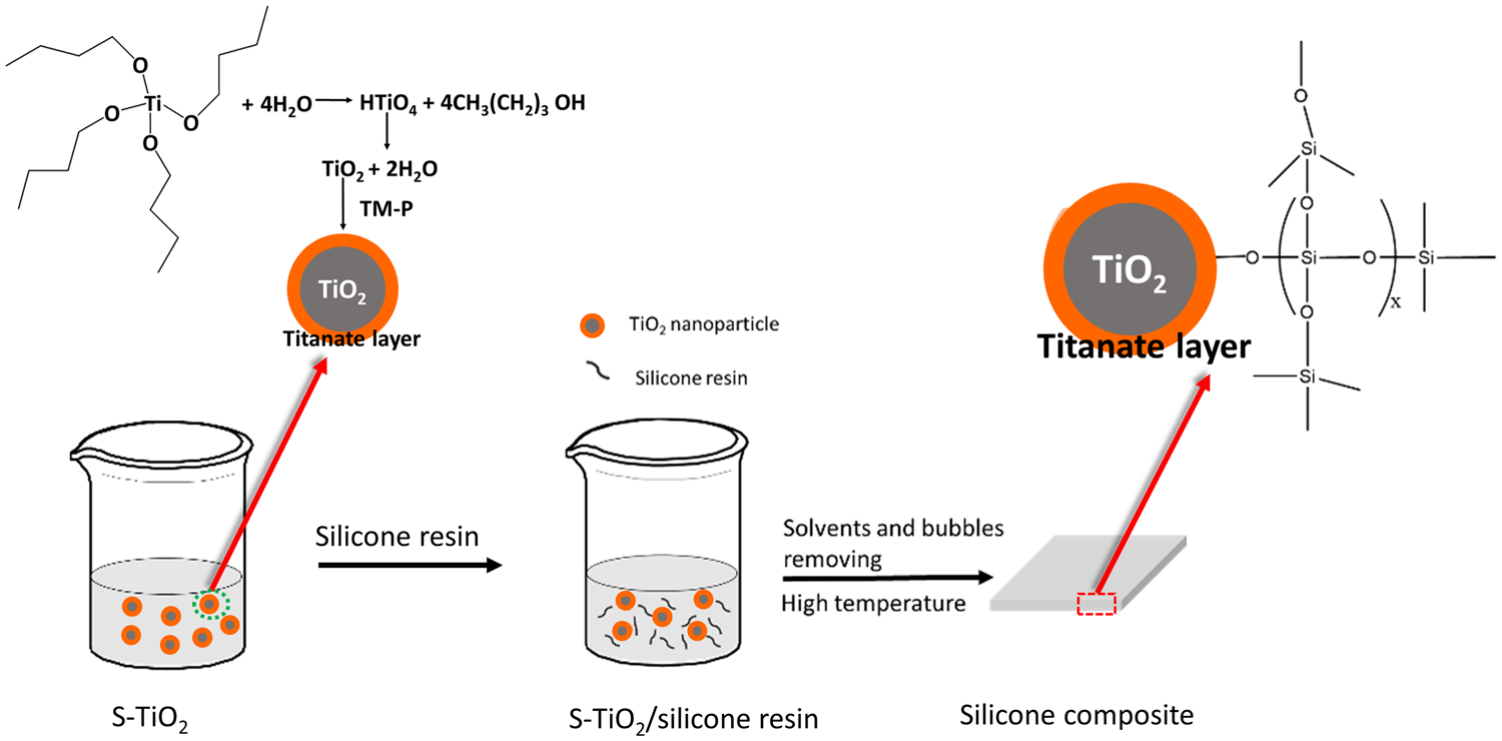
Illustration of the fabrication of S-TiO_2_/silicone nanocomposites.

Figure 1 shows the general steps to fabricate S-TiO_2_/silicone nanocomposites. First, the TiO_2_ nanoparticles synthesized via sol-gel approach were introduced into silicone resin/acetic ether solution under ultrasonic treatment, forming a uniform suspension. Subsequently, the resulting suspension was subjected to a vacuum chamber to remove acetic ether and bubbles. Finally, the transparent nanocomposites were prepared by curing the resin at 100 °C. The only difference of the preparation procedure between S-TiO_2_/silicone nanocomposite and un-treated TiO_2_/silicone nanocomposite is the introduction of titanate coupling agent, which endows TiO_2_ nanoparticles with protective layers on their surface.

**Figure 2:**
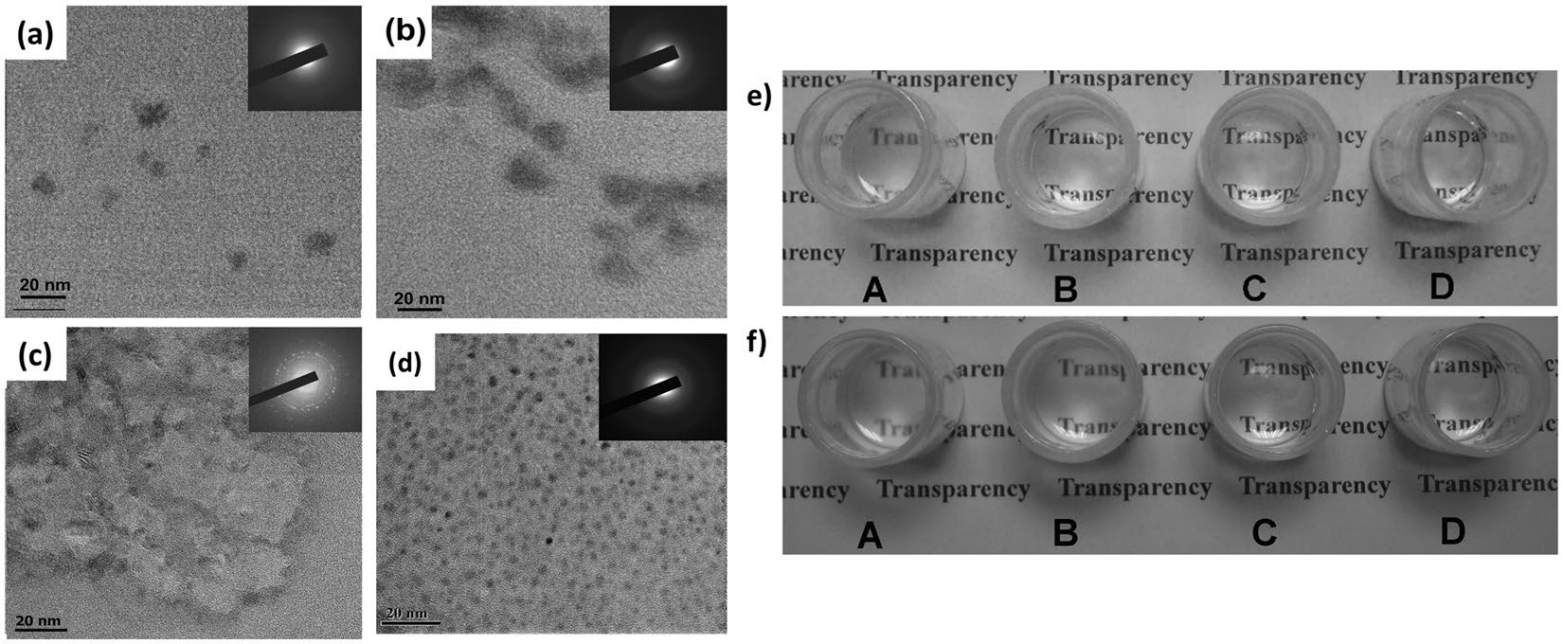
TEM images of (**a**) TiO_2_-1, (**b**) TiO_2_-2, (**c**) TiO_2_-3 and (**d**) S-TiO_2_; solubility of (**e**) S-TiO_2_ and (**f**) silicone resin in (A) acetone, (B) petroleum ether, (C) cyclohexane and (D) acetic ether.

The morphology of the as-prepared TiO_2_ materials was characterized by TEM. As shown in Fig. 2, the TiO_2_-1 has a diameter of approximately 5 nm. Increasing the reaction time from 48 h to 72 h leads to an increase of the diameter of TiO_2_-2 nanoparticles from ca. 10 to ca. 15 nm, accompanying with TiO_2_ aggregation. TiO_2_-3 obtained by drying TiO_2_-2 at 120 °C for 24 h shows a significant increase in the diameter of particles up to the scale of micrometer. By contrast, S-TiO_2_ nanoparticles with a diameter of 3–4 nm are mono-dispersed in the composite. The afore-mentioned results are supported by the BET measurement. As shown in Table S1, specific surface areas of 35.8, 17.6, 2.84, and 75.6 m^2^/g are achieved for TiO_2_-1, TiO_2_-2, TiO_2_-3 and S-TiO_2_, respectively, which agrees well with the result for the diameter of TiO_2_ nanoparticles. X-ray diffraction patterns (top insets of Fig. 2a–d) indicate that the crystallinity of TiO_2_ increases with the increases of reaction time and temperature. Additional evidence on the chemical structure of the TiO_2_ particles was provided by EDS analysis. C originated from titanate coupling agent is observed in S-TiO_2_ while no trace of C is found in TiO_2_-1, TiO_2_-2 and TiO_2_-3 (Table S1), indicating formation of protective layers on the surfaces of S-TiO_2_. Due to the formation of protective layers on their surfaces during the growth of TiO_2_ nanoparticles, the increase of the TiO_2_ diameter is declined and stopped until the formation of intact layers on their surfaces.

### Dispersibility of TiO_2_

To achieve a homogenous dispersion of TiO_2_ in silicone resin, co-solvent is needed to dilute silicone resin due to the poor solubility of silicone resin in ethanol. As shown in Fig. 2e and f, the suspensions of S-TiO_2_ and silicone resin formed in acetic ether both are transparent, while their suspensions formed in other solvents including acetone, petroleum ether and cyclohexane are turbid. In addition, acetic ether featured by low toxicity and low boiling point is a good candidate for the massive fabrication of TiO_2_/silicone nanocomposite. The dispersity of TiO_2_-1, TiO_2_-2 and TiO_2_-3 in acetic ether was also investigated via the same strategy. As presented in Table 1, all of the suspensions of TiO_2_-1, TiO_2_-2 and TiO_2_-3 formed in acetic ether are turbid, indicating that the dispersibility of S-TiO_2_ in acetic ether is much better than that of TiO_2_ without surface modification. In addition, to reveal the effectiveness of various surface modifiers in improving the dispersibility of TiO_2_, the transparency of their suspensions in acetic ether was further studied. As presented in Table S2, all of the suspensions of TiO_2_ modified by oxalic acid, citric acid, KH-550 and KH-560 are turbid, indicating that titanate coupling agent is the most effective in improving the dispersibility of TiO_2_ in acetic ether.

**Table 1:** Dispersibility of various TiO_2_ particles in acetic ether.

	TiO_2_-1	TiO_2_-2	TiO_2_-3	S-TiO_2_
Transparency*	X	X	X	O

^*^Note: Turbid: X, Transparent: O.

### Transmittance of TiO_2_/silicone nanocomposites

**Figure 3:**
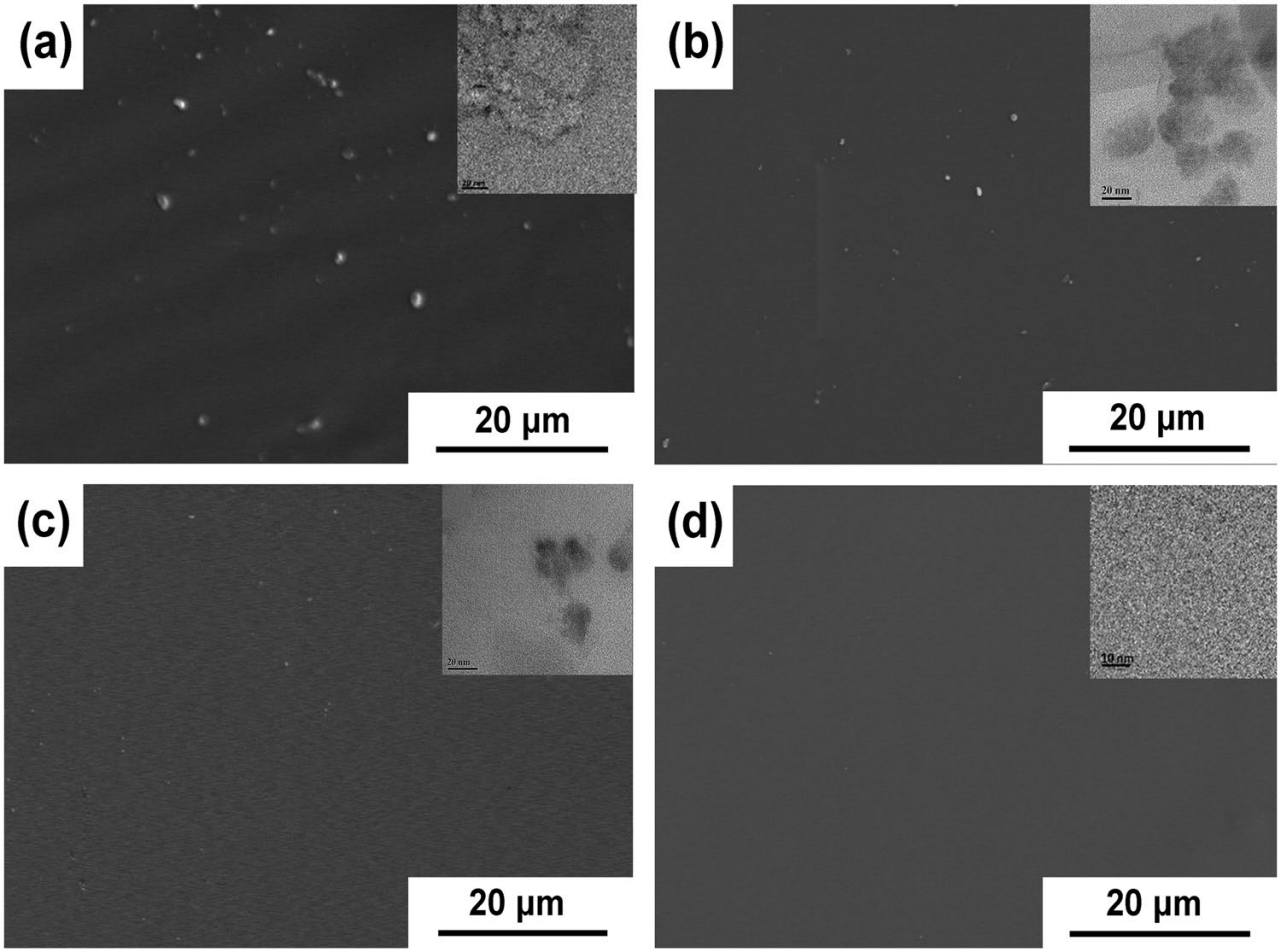
SEM images of the cross sections of silicone nanocomposites with 1 wt% (**a**) TiO_2_-3, (**b**) TiO_2_-2, (**c**) TiO_2_-1, and (**d**) S-TiO_2_ (top inset: TEM image of the corresponding TiO_2_/silicone nanocomposite).

The cross sections of silicone nanocomposites of various TiO_2_ nanoparticles were observed with SEM. As shown in Fig. 3a–c, aggregates sized ca. 150 nm, 30 nm and 10 nm are observed in the nanocomposites incorporated with TiO_2_-1, TiO_2_-2, and TiO_2_-3, respectively, suggesting the poor dispersion of TiO_2_-1, TiO_2_-2 and TiO_2_-3 in the silicone resin. In comparison, no visible aggregates appear in the S-TiO_2_/silicone nanocomposite. S-TiO_2_ nanoparticles are mono-dispersed in the silicone resin with a diameter around 3–4 nm (see the top inset of Fig. 3d). The narrow size distribution and excellent dispersion of S-TiO_2_ in the silicone resin are attributed to the titanate coupling agent layers formed on the surfaces of TiO_2_ nanoparticles, endorsing the excellent compatibility between TiO_2_ nanoparticles and silicone resin, and thus eliminating the aggregation of TiO_2_ nanoparticle in both acetic ether and silicone resin.

**Figure 4:**
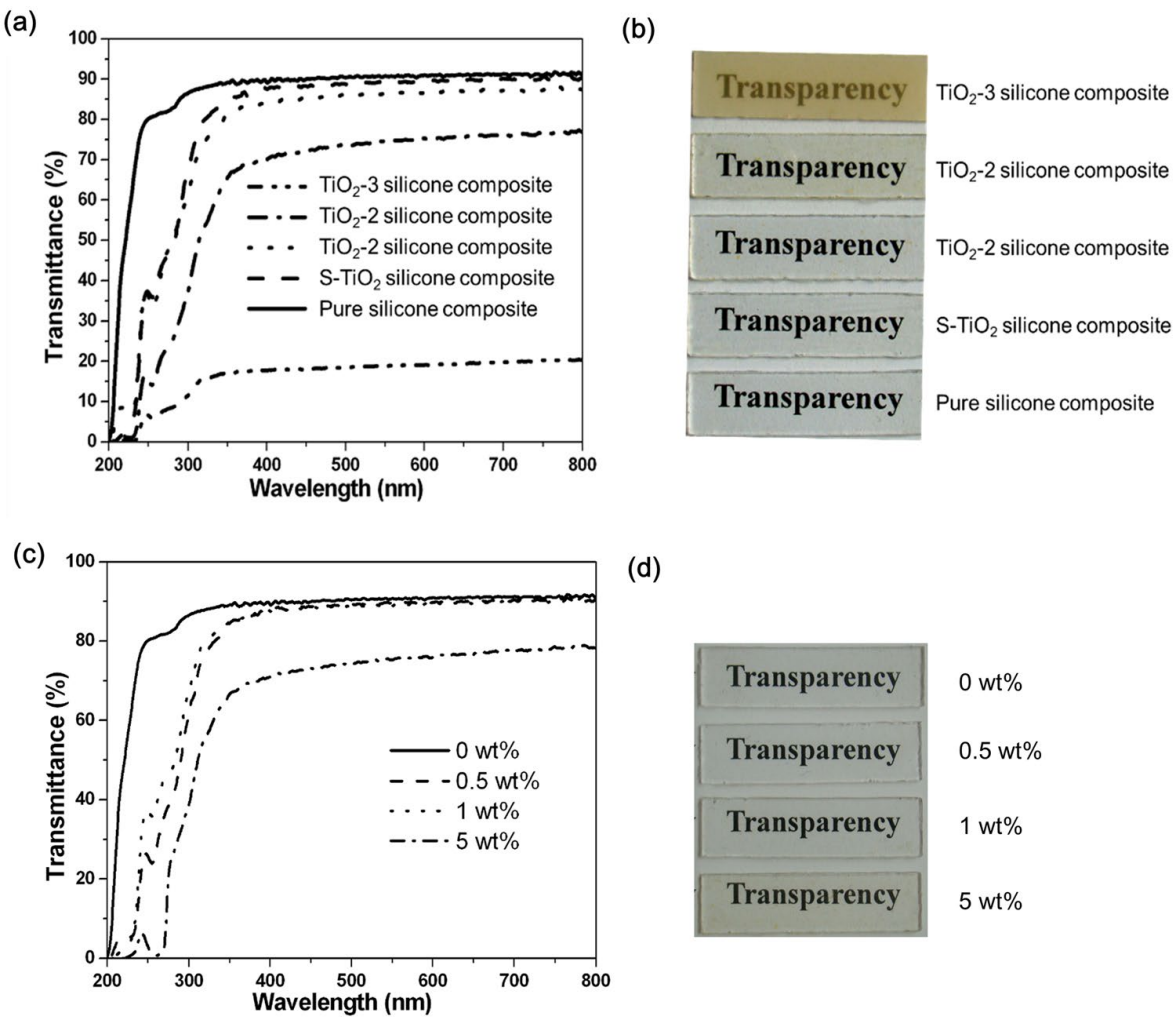
(**a**) Transmittance and (**b**) photograph of the silicone nanocomposite with 1 wt% TiO_2_-3, TiO_2_-2, TiO_2_-1 and S-TiO_2_, and pure silicone as the control; (**c**) transmittance and (**d**) corresponding photograph of titanate S-TiO_2_/silicone nanocomposites with varied S-TiO_2_ mass fractions.

The following equation gives the light intensity of a spherical particulate composite^26, 27^:

1 $$\frac{{\rm{I}}}{{{\rm{I}}}_{0}}=\exp \{[-\frac{32{{\rm{V}}}_{{\rm{P}}}{\rm{x}}{\pi }^{4}{{\rm{r}}}^{3}}{{{\rm{\lambda }}}^{4}}{(\frac{{({{\rm{n}}}_{{\rm{p}}/}{{\rm{n}}}_{{\rm{m}}})}^{2}-1}{{({{\rm{n}}}_{{\rm{p}}/}{{\rm{n}}}_{{\rm{m}}})}^{2}+2})}^{2}]\}$$

where r, n_p_ and V_p_ are the radius, RI and volume fraction of spherical particles, respectively. n_m_ is the RI of matrix, λ is light wavelength, and x is the thickness of the composite. As revealed in Equation 1, the diameter of nano-filler significantly affects the transmittance of the composite. Therefore, to maintain the high transparency of the composite, the ultra-fine nano-fillers with mono-dispersion are strongly required. Meanwhile, the transmittance of a composite is highly related to the thickness of composites, it is quite difficult to achieve a high transparency for bulk materials than thin films. To our best knowledge, all of the transparent composites with enhanced RI reported previously are thin films with a thickness of 10^−8^ to 10^−4^ m, whereas bulk transparent nanocomposites with a thickness of 10^−3^ to 10^−2^ m are rarely reported^27–30^. The thickness of the resin layer as encapsulating material surely has some effect on the lighting efficiency of LED. In this study, the thickness of the as-prepared silicone composite is ca. 1 mm (namely ca. 10^−3^ m), close to that of realistic packaging materials for LEDs. This could give us practical evaluation of the current protocol on the performance of the as-prepared silicone composite. The high specific surface area of nanoparticles would lead to irreversible aggregation^32, 33^. Aggregated nanoparticles usually have much larger sizes than individual nanoparticles, and thus would have an adverse effect on the transparency and lighting efficiency of LED. In this work, S-TiO_2_ nanoparticles are mono-dispersed in the silicone resin, and thus the resultant composite as encapsulating material has a high transparency (Fig. 4). As a result, the introduction of S-TiO_2_ would have no adverse effect on the efficiency of LED.

Figure 4a shows the transmittance of the pure silicone resin and silicone nanocomposites with 1 wt% TiO_2_. Compared with the pure silicone resin, only 2% decrease in transmittance is achieved in the S-TiO_2_/silicone nanocomposite. As the TiO_2_ particle size increases, the transparency of silicone nanocomposite decreases. Figure 4b visually demonstrates the transparency of the corresponding silicone nanocomposites. The transparency of TiO_2_-1/silicone nanocomposite and TiO_2_-2/silicone nanocomposite is approximately 83% and 75%, respectively. Further increase in the particle size of TiO_2_ results in an opaque silicone nanocomposite with 20% of transmittance in the visible light range. This change obeys Equation 1.

The effect of S-TiO_2_ filler content on the transmittance is shown in Fig. 4c. With the addition of 0.5 and 1.0 wt% S-TiO_2_, the transmittance of silicone nanocomposite is close to that of pure silicone resin, and the average decrease of the transmittance in the visible range of 400–800 nm is less than 2%. As revealed in Fig. 4d, no visible transparency difference is seen with naked eyes between the pure silicone resin and the nanocomposites filled with 0.5 and 1.0 wt% of S-TiO_2_. Even increasing the content of S-TiO_2_ up to 5.0 wt%, the transmittance in the visible range still maintains at a high level of approximately 70%, indicating that the surface modification of TiO_2_ with titanate coupling agent is very effective in fabricating highly transparent TiO_2_/polymer nanocomposite.

### RI of TiO_2_/silicone nanocomposites

The lighting efficiency of LED can be determined by the difference of refractive index between lighting chips and packaging materials as indicated by Equation 2 ^31^:

2 $${{\rm{\eta }}}_{{\rm{encapsulated}}}=(\frac{1-\,\cos (\arcsin \frac{{{\rm{n}}}_{{\rm{pm}}}}{{{\rm{n}}}_{{\rm{chip}}}})}{1-\,\cos (\arcsin \frac{1}{{{\rm{n}}}_{{\rm{chip}}}})}){{\rm{\eta }}}_{{\rm{unencapsulated}}}$$

where η_encapsulated_ and η_unencapsulated_ are the lighting efficiency of encapsulated and un-encapsulated LED chip, respectively; n_pm_ and n_chip_ are the refractive index of packaging materials and LED chip, respectively. It is understandable from Equation 2 that as the RI of the packaging material increases, the lighting efficiency first increases quasi-linearly, then sub-linearly, and finally reaches a saturation. Since the RI of conventional lighting chip material is 2.5~3.0, the packaging material with a higher RI will lead to a higher lighting efficiency of LED. In this work, the introduction of fine TiO_2_ particles with a high RI brings about a high RI of the composite as encapsulating material, aiming at increasing the lighting efficiency of LED.

**Figure 5:**
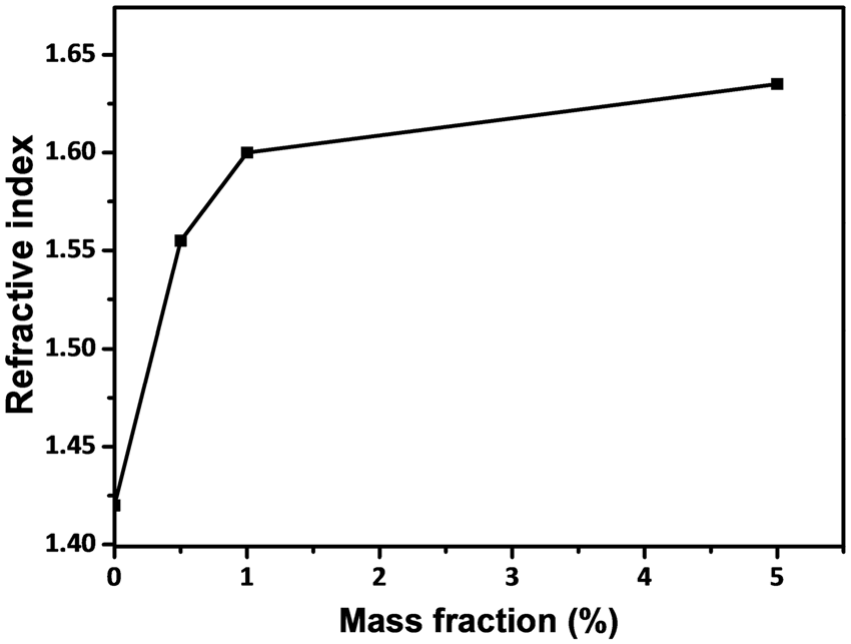
RI of S-TiO_2_/silicone nanocomposites with different mass fractions.

The RI measured for the silicone nanocomposite with 1 wt% S-TiO_2_, TiO_2_-1, TiO_2_-2, and TiO_2_-3 is 1.596, 1.596, 1.602, and 1.642, respectively. The RI of silicone nanocomposite filled with TiO_2_-3 is somewhat higher than other nanocomposites, due to the crystallinity of TiO_2_-3 formed with longer aging time is better than that of S-TiO_2_, TiO_2_-1, and TiO_2_-2. Figure 5 shows the RI of S-TiO_2_/silicone nanocomposites as a function of S-TiO_2_ mass fraction. As the S-TiO_2_ loading increases from 0 to 0.5 and 1 wt%, the RI of silicone resin is dramatically increased from 1.42 to 1.56 and 1.596, achieving the improvement of 9.8% and 12.3%, respectively. In comparison with other researches, the current strategy shows higher improvements in RI. For instance, the incorporation of 1% of silane modified TiO_2_ and oleic acid capped TiO_2_ in to silicone resin increases the RI of silicone composite from 1.51 to 1.56 and 1.575, respectively^32, 33^. As the content of S-TiO_2_ increases further, the increasing rate of the RI is declined. Less than 2% of increase in RI is achieved as the content of S-TiO_2_ increases further to 5 wt%, indicating that the effectiveness of RI enhancement at a higher filler content is limited. In principle, the porosity if any would reduce the RI of the composite since the air has a RI of unity. In the current work, the porosity measured on the surfaces of TiO_2_ particles was around 3%. On the other hand, silicone resin might penetrate into the porosity. As a result, the effect of the porosity on the RI could be neglected. Therefore, the RI of the composite was determined mainly by TiO_2_ particles and enhanced by the incorporation of TiO_2_ particles as confirmed by Fig. 5.

### Thermal stability of TiO_2_/silicone nanocomposite

**Figure 6:**
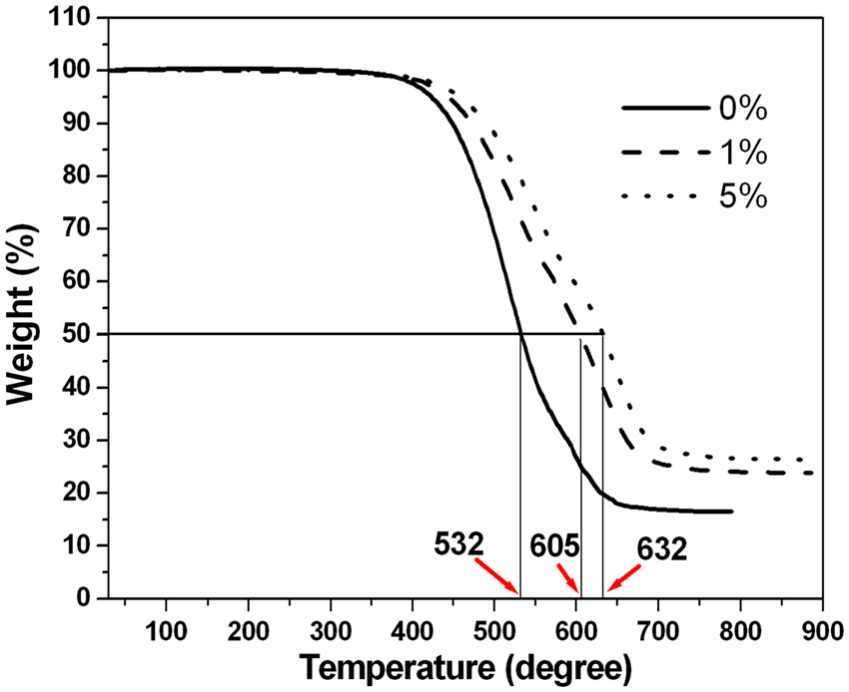
TGA curves of pure silence resin and S-TiO_2_/silicone nanocomposites with 1 wt% and 5 wt% S-TiO_2_.

Figure 6 shows the TGA curves of the pure silicone resin and the silicone nanocomposites with various S-TiO_2_ loadings. Although the starting decomposition temperature is almost the same for the pure silicone resin and the silicone nanocomposites, the incorporation of TiO_2_ nanoparticles decreases the decomposition speed of the silicone nanocomposite. Taking the example of the weight loss of 50%, the temperature of the silicone nanocomposites with 0%, 1% and 5% S-TiO_2_ is 532, 605 and 632 °C, respectively; namely, the temperature corresponding to the weight loss of 50% has been increased by 73 and 100 °C, respectively. The “cross-link density” of the nanocomposites is increased with the incorporation of TiO_2_ nanoparticles, which leads to the enhancement of their thermal stability with a decreased decomposition rate^34^.

## Conclusions

In summary, a facile approach to produce highly transparent TiO_2_/silicone nanocomposite with a high RI and a high thermal stability has been demonstrated by the incorporation of S-TiO_2_ nanoparticles treated by titanate coupling agent into diluted silicone resin via simple solvent mixing. The titanate coupling agent layers formed on the surfaces of ultra-fine 3–4 nm TiO_2_ nanoparticles endorses the excellent dispersion of S-TiO_2_ nanoparticles in the silicone resin and an excellent compatibility between the TiO_2_ nanoparticles and the silicone resin. Consequently, with the addition of 1 wt% S-TiO_2_, the as-prepared silicone nanocomposite shows an enhanced RI from 1.42 to ca. 1.60 (13% enhancement), a high transmittance of 88% in the entire visible light range for 1 mm thick samples and meanwhile an enhanced thermal stability. The current strategy is cost-effective, environmental-friendly, and easy-processing, which has a great potential to be widely used in producing transparent packaging materials with the greatly enhanced RI and meanwhile a high thermal stability.

## Materials and Methods

### Materials

Tetrabutyl titanate (C_16_H_36_TiO_4_) was purchased from Beijing Xingjin Chemical Reagent Company. Absolute ethanol, acetic ether, acetone, petroleum ether, and cyclohexane were supplied by Beijing Chemistry Reagent Company and used as received. Titanate coupling agent (TM-P) was bought from JiangSu YiZheng TianYang Chemical Plant. Silicon resin (KMT-1252) with two parts A and B was purchased from Beijing KMT Technology Co., Ltd.

### Synthesis of S-TiO_2_ nanoparticles

S-TiO_2_ nanoparticles were prepared with a typical sol-gel procedure. First, 0.01 M tetrabutyl titanate was added to 50 mL ethanol, then the solution was stirred for 15 min to ensure complete dissolution of tetrabutyl titanate. Afterwards, 0.12 mL TM-P was introduced into the suspension and stirred for another 15 min. Subsequently, 1 mL distilled water was added to the suspension obtained and kept stirring for 48 h at 40 °C.

In parallel, TiO_2_ nanoparticles with different sizes were synthesized using the similar protocol in the absence of the titanate coupling agent TM-P. The samples react for 48 h and 72 h, denoted as TiO_2_-1 and TiO_2_-2, respectively. TiO_2_-3 was prepared by vacuum drying of TiO_2_-2 at the temperature of 120 °C for 24 h.

### Fabrication of TiO_2_/silicone nanocomposites

In a typical fabrication procedure, 10 g silicon resin with the mass ratio of 10:1 for Part A and Part B was dissolved in 20 mL acetic ether by ultrasonic treatment until a transparent solution was obtained. Then, 0.11 g TiO_2_ particles (TiO_2_-1, TiO_2_-2, TiO_2_-3 or S-TiO_2_) were homogenously mixed with silicone resin/acetic ether solution by ultrasonic treatment. The bubbles and acetic ether were removed by vacuum pump. Finally, the resultant suspension was casted into a stainless steel mould and then transferred into an oven at 100 °C for 2 h. The thickness of as-prepared silicone composites is approximately 1 mm.

### Characterizations

The transmittance of the nanocomposite was obtained from a Hitachi U-3900. The RI was measured on an Abbe Refractometer (WAY-2S) in ambient atmosphere. Transmission electron microscopy (TEM) images and electronic diffraction were taken on a JEM-2100F instrument (operated at 200 kV). Scanning electron microscopy (SEM) images and energy dispersive spectroscopy (EDS) were collected on a Hitachi S-4300. The Brunauer–Emmett–Teller (BET) specific surface areas were calculated using adsorption data in P/P0 = 0.05–0.3 (six points collected). The weight loss analysis of the samples was conducted on a Netzsch STA 409 PC/PG at the rate of 10 °C/min under nitrogen atmosphere. Fourier transform infrared (FTIR) spectra were recorded using a Varian 3100 FT-IR spectrometer with 2 cm^−1^ resolution and accumulation of 24 scans.

## Electronic supplementary material

Supporting information
