# Supplementary material for: High performance surface-modified TiO2/silicone nanocomposite
Source: Sci Rep. 2017 Jul 20;7:5951. doi: 10.1038/s41598-017-05166-7 (PMC5519632; doi:10.1038/s41598-017-05166-7)
Supplement: Supplementary file 1 — Supporting information [file 41598_2017_5166_MOESM1_ESM.doc]

**High performance surface-modified TiO_2_/silicone nanocomposite**

**Pei Huang,^†, §^ Han-Qiao Shi,^ζ,⊥,§^ Hong-Mei Xiao,^⊥^ Yuan-Qing Li,^†^,* Ning Hu^†^ & Shao-Yun Fu,^†,⊥^,***

**Table 1.** Specific surface area and EDS analysis of TiO_2_ originated from different resources

| **Sample** | **TiO_2_-1** | **TiO_2_-2** | **TiO_2_-3** | **S-TiO_2_** |
| --- | --- | --- | --- | --- |
| Specific surface area (m^2^/g) | 35.8 | 17.6 | 2.84 | 75.6 |
| C (wt.%) | 0 | 0 | 0 | 26.6% |

**Table 2.** Dispersibility of TiO_2_ nanoparticles with different surface modifiers in acetic ether.

| **Modifier** | **oxalic acid** | **citric acid** | **KH-550** | **KH-560** | **Titanate coupling agent** |
| --- | --- | --- | --- | --- | --- |
| **Transparency*** | X | X | X | X | O |

*Note: Turbid: X, Transparent: O.
